# Identification of metabolic biomarkers in patients with type 2 diabetic coronary heart diseases based on metabolomic approach

**DOI:** 10.1038/srep30785

**Published:** 2016-07-29

**Authors:** Xinfeng Liu, Jian Gao, Jianxin Chen, Zhiyong Wang, Qi Shi, Hongxue Man, Shuzhen Guo, Yingfeng Wang, Zhongfeng Li, Wei Wang

**Affiliations:** 1 Department of Chemistry, Capital Normal University, Beijing 100048, China; 2 Beijing University of Chinese Medicine, Beijing 100029, China

## Abstract

Type 2 diabetic coronary heart disease (T2DM-CHD) is a kind of serious and complex disease. Great attention has been paid to exploring its mechanism; however, the detailed understanding of T2DM-CHD is still limited. Plasma samples from 15 healthy controls, 13 coronary heart disease (CHD) patients, 15 type 2 diabetes mellitus (T2DM) patients and 28 T2DM-CHD patients were analyzed in this research. The potential biomarkers of CHD and T2DM were detected and screened out by ^1^H NMR-based plasma metabolic profiling and multivariate data analysis. About 11 and 12 representative metabolites of CHD and T2DM were identified respectively, mainly including alanine, arginine, proline, glutamine, creatinine and acetate. Then the diagnostic model was further constructed based on the previous metabolites of CHD and T2DM to detect T2DM-CHD with satisfying sensitivity of 92.9%, specificity of 93.3% and accuracy of 93.2%, validating the robustness of ^1^H NMR-based plasma metabolic profiling to diagnostic strategy. The results demonstrated that the NMR-based metabolomics approach processed good performance to identify diagnostic plasma biomarkers and most identified metabolites related to T2DM and CHD could be considered as predictors of T2DM-CHD as well as the therapeutic targets for prevention, which provided new insight into diagnosing and forecasting of complex diseases.

T2DM, a major public health problem in huge population worldwide, acts as a potent and independent risk factor for several forms of cardiovascular disease (CVD)^1^. Coronary heart disease (CHD) has been recognized as the most common and costly vascular complication of T2DM^2^,^3^,^4^. Because of the intimate correlation between T2DM and CHD, it has been speculated that there are common pathogenic processes and metabolic defects for them. The increasing risk of CHD in T2DM-CHD patients represents one of the main causes for the mortality in this population^5^,^6^. Undoubtedly a delayed recognition of T2DM undoubtedly worsens the prognosis for survival of many T2DM-CHD patients.

In clinical practice, higher levels of cholesterol in large, triglyceride-rich lipoprotein particles, mainly very low-density lipoprotein (VLDL) and low-density lipoprotein (LDL), and lower levels of cholesterol in high-density lipoprotein (HDL) particles are known to be associated with increasing risk of CHD^7^,^8^. However, the risk factors identified so far from cross-sectional epidemiological studies are insufficiently powerful to provide a clinically useful diagnosis of CHD. Diagnosis of T2DM is typically determined by fasting blood glucose and the oral glucose tolerance test that examines an individual’s ability to dispose of a glucose load. Glycated hemoglobin (HbA1c) provides information on glucose management during the months preceding the initial testing^9^,^10^. Patients with T2DM-CHD always sustain a worse prognosis for the survival than patients with CHD or T2DM individually^11^,^12^,^13^. It is clear that single alteration in traditional risk factors (e.g., raised blood pressure, abnormal lipids) cannot explain the excess incidence of CVD in patients with T2DM-CHD. According to the comparison among studies about T2DM and CHD, it could be demonstrated that these two diseases are directly associated with metabolism disorder^14^, but the detailed understanding of serum metabolites in T2DM-CHD is still limited, let alone the diagnosis of T2DM-CHD. Applying novel technology to study serum metabolome may provide useful information to elucidate the mechanism in the development of T2DM-CHD.

Metabonomics, a postgenomic approach used to rapidly identify global metabolic changes in biological systems, has been increasingly applied to diagnose diseases, measure the response to treatment, discover biomarkers and identify perturbed pathways^15^,^16^,^17^. Nuclear magnetic resonance (NMR) spectroscopy is a rapid, non-destructive and high-throughput analytical method and has been widely used in metabonomic research^18^,^19^,^20^,^21^,^22^. It has been reported that NMR-based metabolomic approaches instituting a sensitive high-throughput molecular screening have already demonstrated promising results in diagnosing a variety of diabetes mellitus and cardiovascular system disorders^23^,^24^,^25^.

In this study, we made a novel attempt to explore the potential biomarkers related to CHD and T2DM and validate these potential biomarkers as predictors to diagnose the patients with T2DM-CHD based on the NMR non-targeted metabolomics. Serum samples from T2DM and CHD patients were analyzed by NMR metabolic profile, principle component analysis (PCA) and partial least squares discriminant analysis (PLS-DA) to screen out potential biomarkers. ROC curve analysis for the logistic regression model was constructed by the biomarkers of T2DM and CHD patients for T2DM-CHD prediction. This process may accelerate the advancement in understanding the mechanism of T2DM-CHD occurrence and progression at the metabolic level and providing information for the prediction of early marker metabolites for T2DM-CHD.

## Results

### Demographics and Clinical Characteristics

Detailed data about patients and controls are presented in Table 1. There was no significant difference in gender, age, Body Mass Index (BMI), Systolic Blood Pressure (SBP), Diastolic Blood Pressure (DBP), total cholesterol, Blood Urea Nitrogen (BUN) and serum creatinine (SCr) among the four groups based on SPSS analysis (*p* > 0.05). The level of triglycerides and HbA1c in T2DM and T2DM-CHD was higher than that of the controls (*p* < 0.05). Apart from HDL in T2DM (p > 0.05), HDL in other two groups were higher than that in the controls (*p* < 0.05). As expected, FPG, 2 h plasma glucose (2hPG) and fasting insulin (FINS) in T2DM-CHD and T2DM were higher compared to the controls (*p* < 0.05), particularly in T2DM-CHD (*p* < 0.0001). The level of LDL in CHD and T2DM were a little higher than those in healthy subjects (HC), perhaps due to the influence of the medication such as stains and insulin. Therefore, the findings cannot be attributed to demographic factors.

### ^1^H-NMR analysis of Plasma samples

**Figure 1:**
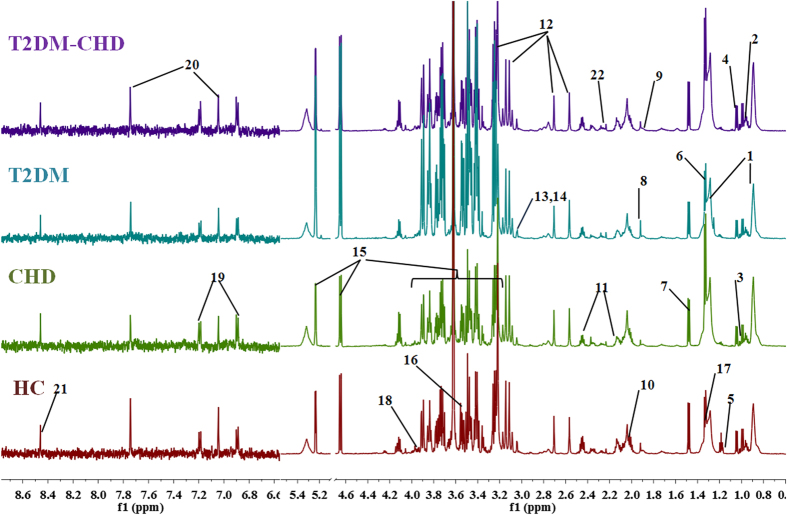
Representative 600 MHz ^1^H NMR spectra of plasma samples from the patients of CHD, T2DM and T2DM-CHD and healthy control subject. Distinguished metabolites: 1 VLDL/LDL, 2 leucine, 3 isoleucine, 4 valine, 5 isopropanol, 6 lactate, 7 alanine, 8 acetate, 9 arginine, 10 proline, 11 glutamine, 12 EDTA, 13 creatine, 14 creatinine, 15 glucose, 16 glycine, 17 threonine, 18 serine, 19 tyrosine, 20 3-methylhistidine, 21 formate, 22 acetone.

Plasma contains almost all of the low molecular weight species in whole blood and a few high molecular weight compounds, thus it can provide valuable bio-information in the organism’s metabolism. Figure 1 shows representative 600 MHz ^1^H NMR CPMG spectra of plasma from the healthy controls, T2DM group, CHD group and T2DM-CHD group. The plasma NMR spectra were dominated by LDL/VLDL (δ0.86, δ1.26), leucine (δ0.95, δ0.97), valine (δ1.03), lactate (δ1.33, δ4.12), alanine (δ1.48), acetate (δ1.92), glucose (δ3.2–4.0, 4.66, 5.23) etc. Resonance assignments were performed according to the existing literatures^26^,^27^,^28^ and in-house NMR database and further confirmed with analysis of the 2D NMR spectroscopy (the spectra was shown in Fig. S1). Visual inspection of the ^1^H NMR spectra showed subtle differences in plasma metabolites between groups. In the ^1^H NMR spectral of plasma samples, the dominated change of the signals among low molecular weight metabolites like leucine, isoleucine, valine, alanine, glutamine, creatine, proline, glucose etc. were detected. Multivariate data analysis was further performed to obtain more detailed analysis of metabolic differences between groups.

### Multivariate data analysis and the selection of potential biomarkers

**Figure 2:**
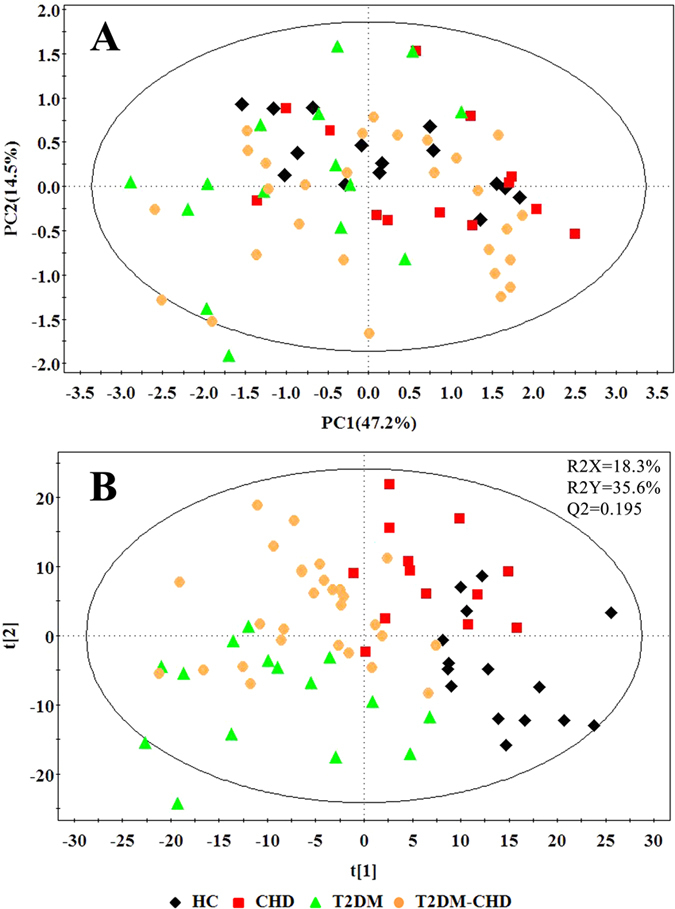
PCA and PLS-DA score plot of HC, CHD patients, T2DM patients and T2DM-CHD patients.

PCA was used for the overview of the metabonomic data set and the spotting of outliers, and then for the detection of any grouping. This type of analysis is designed to highlight systematic variation across series of NMR spectra. It results in the calculation of a series of principal components (PCs) for each sample. The PCA scores plot was used to reveal observations lying outside the 0.95 Hotelling’s T2 ellipse. The score plot was obtained with the first two PCs presenting 47.2% and 14.5% variance, respectively (Fig. 2A). PLS-DA model was established to investigate the metabolic differences between four groups. The PLS-DA score plot displayed a good separation between HC group and other disease groups (Fig. 2B).

**Figure 3:**
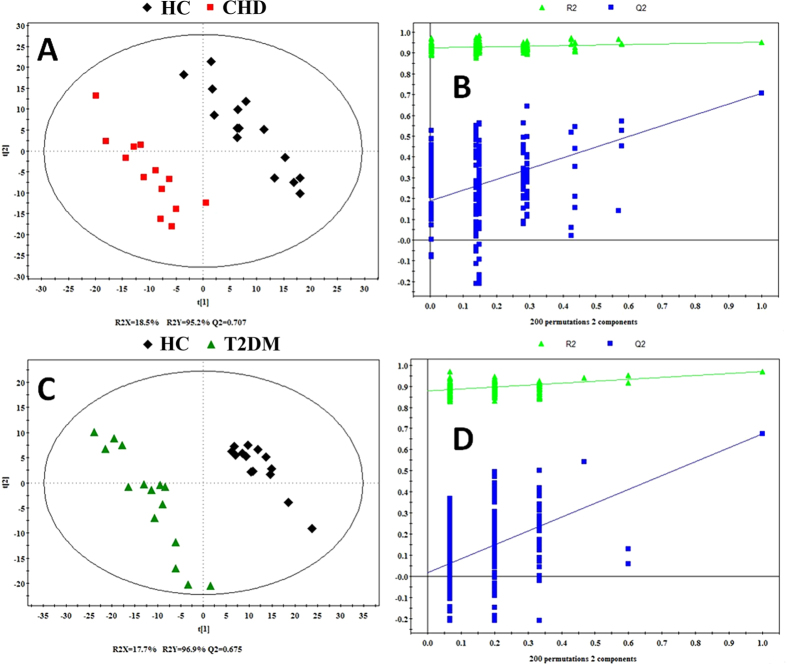
PLS-DA score plot (**A,C**) of CHD and T2DM patients and HC and statistical validation of the PLS-DA (**B,D**). Score plots showed the degree of separation of the model between CHD **(red boxes) and HC (black Diamonds)** (R^2^X = 18.5%, R^2^Y = 95.2%, and Q^2^ = 0.707), and T2DM patients (**green triangles**) and HC (**black Diamonds**) (R^2^X = 17.7%, R^2^Y = 96.9%, and Q^2^ = 0.675). A permutation test performed with 200 random permutations in a PLS-DA model showing R^2^ (green triangles) and Q^2^ (blue boxes) values from the permuted analysis (bottom left) significantly lower than the corresponding original values (top right).

Then, both of the two PLS-DA models with satisfactory discriminating ability were established to assess the metabolic differences between two disease groups (CHD and T2DM) and HC group respectively (Fig. 3). According to the score plot of the PLS-DA model, CHD patients and HC were discriminated obviously with R^2^X = 18.5%, R^2^Y = 95.2%, and Q^2^ = 70.7% (Fig. 3A), and the T2DM patients and HC were discriminated with R^2^X = 17.7%, R^2^Y = 96.9%, and Q^2^ = 0.675 (Fig. 3C). The parameters for describing the PLS-DA models were significantly elevated (R^2^Y, Q^2^ > 0.5), which suggested that the PLS-DA models were robust^29^. The validation plot (Fig. 3B,D) demonstrated that the original PLS-DA models were not random and overfitting as both permutated Q^2^ and R^2^ values were significantly lower than the corresponding original values.

**Figure 4:**
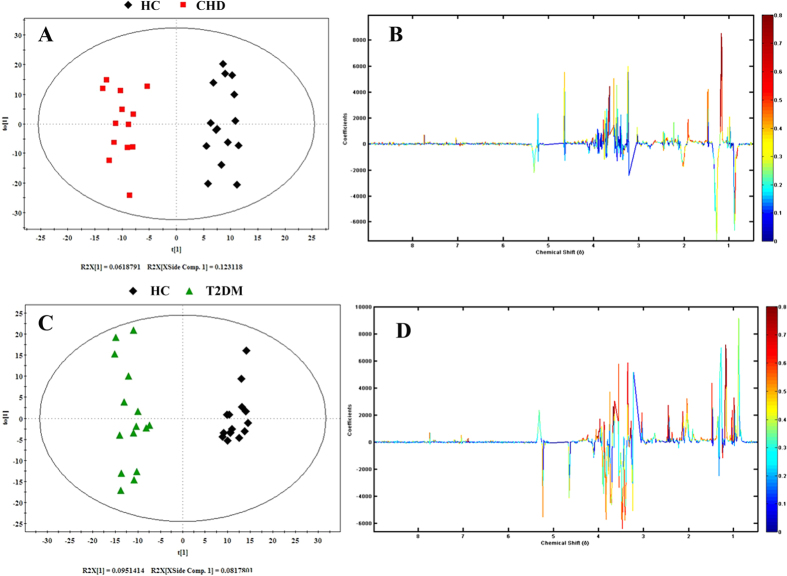
OPLS-DA score plot and corresponding color-coded correlation coefficient loading plots of CHD and T2DM patients and HC. This figure displayed the degree of separation of the model between CHD **(red boxes) and HC (black Diamonds)** (R^2^X = 18.5%, R^2^Y = 95.2%, and Q^2^ = 0.462) and OPLS-DA score plot (**C**) of T2DM patients and healthy controls (HC), displaying the degree of separation of the model between T2DM (**green triangles**) and HC (**black Diamonds**) (R^2^X = 17.7%, R^2^Y = 96.9%, and Q^2^ = 0.622); OPLS-DA Corresponding color-coded correlation coefficient loading plots (**B**,**D**) of key metabolites, demonstrating discrimination of key metabolite levels between CHD and T2DM patients and HC.

In order to eliminate the influence of individual difference and conduct an insight into the changed metabolites responsible for the separation between two groups, the OPLS-DA model was constructed using the first principal component and the first orthogonal component. In Fig. 4, it reveals the OPLS-DA score plots for pairwise comparison of CHD, T2DM and HC group samples, along with the corresponding coefficients plots depicting the major discriminators. In the score plot (Fig. 4A, R^2^Y = 95.2%, Q^2^ = 0.462), a significant biochemical distinction between the CHD patients and HC was identified and there was also a significant biochemical distinction between the T2DM patients and healthy controls in the score plot (Fig. 4C, R^2^Y = 96.9%, Q^2^ = 0.622). The metabolic changes in patients were reflected in the color coded coefficient plots (Fig. 4B,D). Metabolites exhibiting significant changes (p < 0.05) were identified based on the absolute cutoff value of correlation coefficients (|r|) and VIP value and were listed in Table 2. The resonances assigned to proline and creatine were significantly increased, but the levels of isopropanol, alanine, leucine, arginine, acetate, glutamine, glycine, glucose and 3-methylhistidine were statistically decreased in the CHD group compared to those of the HC group. The T2DM group had lower levels of isoleucine, leucine, valine, isopropanol, alanine, arginine, glutamine, proline, creatinine, threonine and tyrosine, but had higher levels of glucose compared to thoes of the HC group. The potential biomarkers related to T2DM and CHD screened out above were used to predict the process and mechanism of T2DM-CHD.

### Hierarchical cluster analysis (HCA) of biomarkers for T2DM-CHD diagnosis

**Figure 5:**
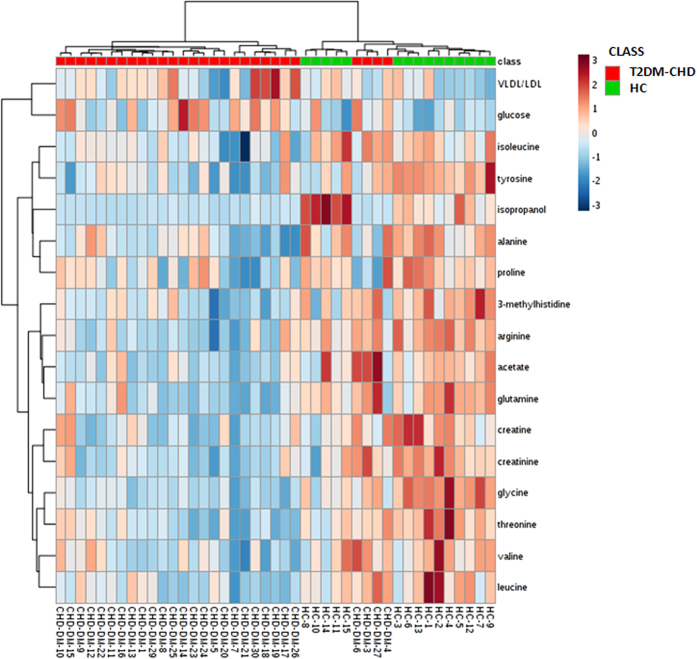
Heatmap visualization based on 17 biomarkers. Rows: samples; columns: biomarkers. Green: HC; red: T2DM-CHD patients. Color key indicates metabolite expression value: dark blue: lowest; dark red: highest.

HCA could readily be used to assess relatedness and distance of any type of samples characterized by any type of descriptors, and the result was displayed as ‘heatmap’. We used the metabolites listed in Table 2 as the variables to conduct the HCA, and got the heatmap (Fig. 5). From the heatmap, the similarity of different metabolites and different samples could be shown visually. The heatmap showed that the T2DM-CHD patients and healthy controls were almost completely separated from each other. It could be observed that the metabolic state of T2DM-CHD patients resulted in the decreased levels of isopropanol, glycine, alanine, arginine, proline, glutamine, acetate, creatine, 3-methylhistidine, creatinine, isoleucine, tyrosine, valine, threonine and leucine, as well as elevated levels of VLDL/LDL and glucose. The result of HCA further illustrated that these metabolites could distinguish the T2DM-CHD patients and HC, so these endogenous metabolites could be used as the potential biomarkers.

### Prediction and the diagnostic test to the T2DM-CHD disease

The 17 potential metabolites responsible for discrimination between T2DM-CHD patients and HC were identified. Table 3 shows the variation of the integrals of the normalized spectral regions responsible for these 17 metabolites and lists the results from the student’s t-test (p < 0.05) for comparison of HC and T2DM-CHD.

**Figure 6:**
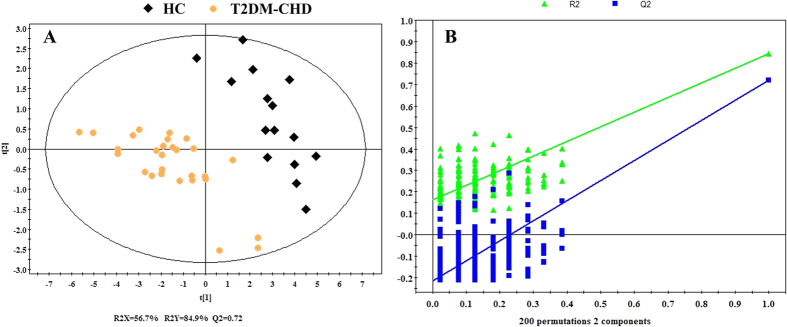
PLS-DA score plot (**A**) and Statistical validation of T2DM-CHD and HC. Score plots (**A**) showing the degree of separation of the model between T2DM-CHD **(orange dots)** and HC **(black Diamonds)** (R^2^X = 56.7%, R^2^Y = 84.9%, and Q^2^ = 0.72) and Statistical validation of the PLS-DA (**B**). A permutation test performed with 200 random permutations in a PLS-DA model showing R2 (green triangles) and Q2 (blue boxes) values from the permuted analysis (bottom left) significantly lower than the corresponding original values (top right).

As is shown in Fig. 6A, a complete separation of T2DM-CHD patients and HC in PLS-DA score plots based on the 17 potential metabolites (R^2^X = 56.7%, R^2^Y = 84.9, Q^2^ = 0.72), suggesting a severe metabolic disturbance of the 17 potential metabolites in T2DM-CHD patients by a supervised PLS-DA with a well goodness of fit (displayed in Fig. 6B).

**Figure 7:**
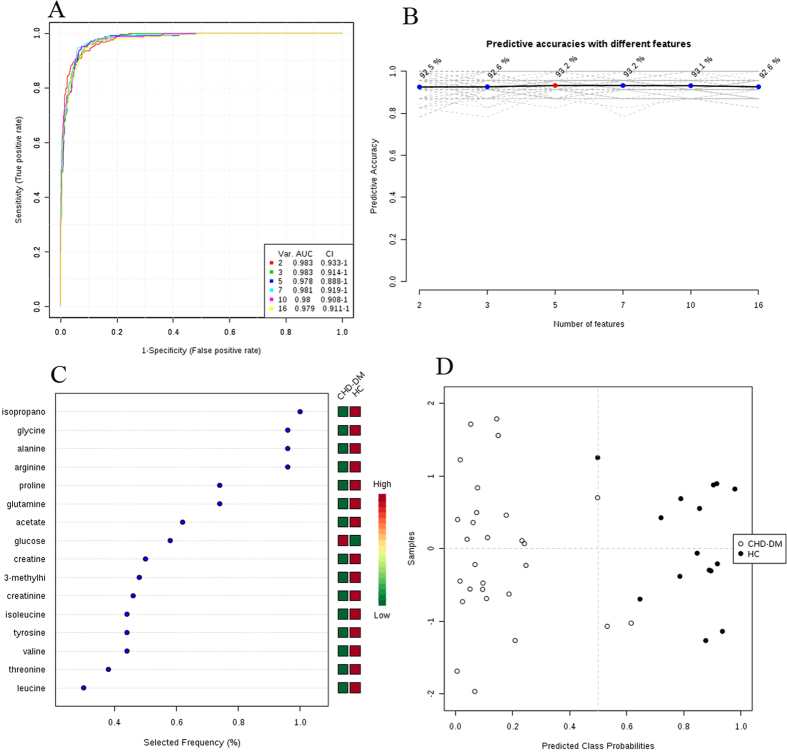
Comparison of different variables based on ROC curves (**A**), the legend shows the feature numbers and the AUCs of the six models, the predictive accuracies (**B**) with different features based on ROC curves, the average importance (**C**) of the 17 metabolites based on ROC curves, Variable Importance in Projection (VIP) plot indicating the most discriminating metabolite in descending order of importance, and (**D**) Prediction of UAP patients and control using MCCV analysis. The class membership of the left-out sample was predicted using an a priori cut-off value of 0.5 (dashed line).

Then, ROC curves analysis was performed to validate the clinical effect of these potential biomarkers in diagnosing the T2DM-CHD. Areas under the ROC curve (AUC) were generally considered as the method of choice for evaluating the performance of potential biomarkers: the greater the AUC, the better the prediction of the model. In Fig. 7A, it showed a set of ROC curves for SVM models created using different subsets of metabolites selected by the filter approach, and six models were developed. The top 2 important variables (isopropanol and glycine) were used to build classification models, the AUC value was 0.983 and 95% confidence interval (CI) was 0.933~1. The AUC using a larger number of variables tried to achieve even greater areas under the ROC curves, and the maximum value was 0.983 (95% CI, 0.933~1) when we used 2 or 3 metabolites as the variables. Meanwhile, the predictive accuracy was the maximum value 93.2% when we use 5 or 7 metabolites as the variables (Fig. 7B). The metabolites in Fig. 7C were ranked by their contribution to distinguish the T2DM-CHD from HC. The greater the distance from the Y-axis, the greater the contribution of a particular metabolite in distinguishing cases from controls. This plot also indicated whether the metabolite concentration was increased or decreased in cases related to controls. The metabolites in Fig. 7C included isopropanol, glycine, alanine, arginine, proline, glutamine, acetate, glucose, creatine, 3-methylhistidine, creatinine, isoleucine, tyrosine, valine, threonine and leucine, and the importance decreased in this order, while the VLDL/LDL was rejected as it made little contribution to distinguish the T2DM-CHD and HC. The predicted class probabilities (average of the cross-validation) for each sample using the best classifier (based on AUC) is illustrated in Fig. 7D. The verification results showed that in the 28 T2DM-CHD samples, 26 were predicted correctly, and in the 15 HC samples, 14 were predicted correctly. Therefore, the OPLS-DA prediction model exhibited a sensitivity of 92.9% and a specificity of 93.3% for T2DM-CHD diagnosis. On the basis of selected biomarkers, ROC analysis revealed that T2DM-CHD could generate signature biomarkers and in return these biomarkers could be used to diagnose them.

### Metabolic Pathway and Function Analysis

**Figure 8:**
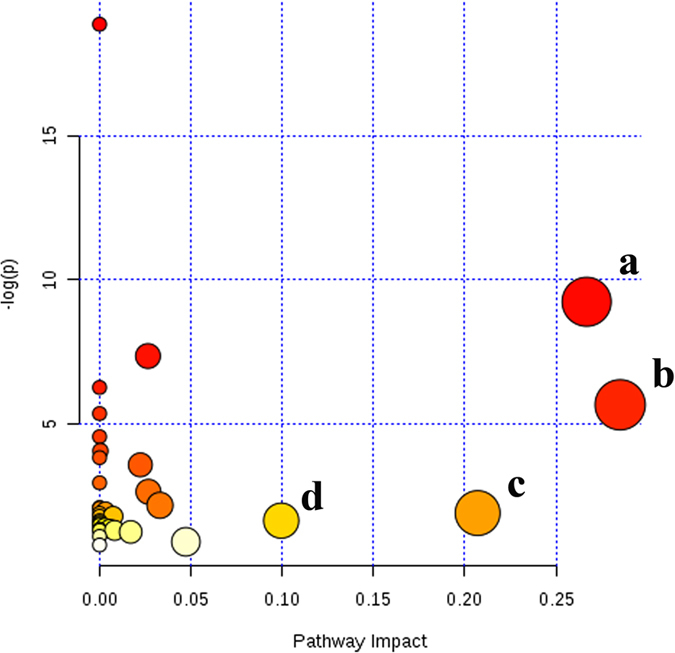
Summary of pathway analysis with MetPA. (**a**) Arginine and proline metabolism (**b**) Glycine, serine and threonine metabolism, (**c**) Alanine, aspartate and glutamate metabolism, (**d**) Pyruvate metabolism.

In addition, based on the identified biomarkers, the plasma metabolic pathway analysis was performed using MetPA software to reveal the most relevant pathways related to T2DM-CHD. The impact value of these pathways calculated from pathway topology analysis above 0.1 was screened out as potential target pathway. According to the impact value, finally there were 4 potential target pathways related to 8 metabolites identified in this research. There were 4 pathways disturbed when T2DM-CHD occurred (Fig. 8), including arginine and proline metabolism, Glycine, serine and threonine metabolism, alanine, aspartate and glutamate metabolism and Pyruvate metabolism, which included more than one target. The details of pathways were displayed in supplementary Table S1 and Figures S2–S5, Supporting Information.

## Discussion

The development of CHD and T2DM in patients is a serious problem that compromises the quality of life and survival of patients. Taking into account of the tendency to population aging observed during the last years, the problem of T2DM-CHD has become even more serious. The precise mechanism linking between CHD and T2DM is not completely clear and there are still unknown factors. Biomarkers predicting T2DM-CHD are useful to identify individuals at high risks of developing T2DM-CHD. Metabolomics is increasingly being applied towards the identification of biomarkers for disease diagnosis, prognosis and risk prediction.

In the present study, ^1^H NMR-based metabonomic approach was conducted to demonstrate metabolic differences between HC and T2DM-CHD. Subsequent analysis of the metabolite profiles of serum samples from CHD and T2DM patients could distinguish patients from healthy normal controls and provide a fingerprint of metabolic changes that characterized the disease, and highlighted the potential of metabolomic analysis in the evaluation of a disease condition. About 17 metabolic biomarkers were highly possible to be associated with T2DM-CHD, which showed better performance in terms of both specificity and sensitivity. These metabolites included isoleucine, valine, isopropanol, alanine, leucine, acetate, proline, glutamine, arginine, trans-aconitate, creatine, creatinine, glucose, glycine, threonine, tyrosine and 3-methylhistidine. The diagnostic model using ROC curves was further constructed based on the metabolites of CHD and T2DM to predict T2DM-CHD with satisfying sensitivity of 92.9%, specificity of 93.3% and accuracy of 93.2%.

**Figure 9:**
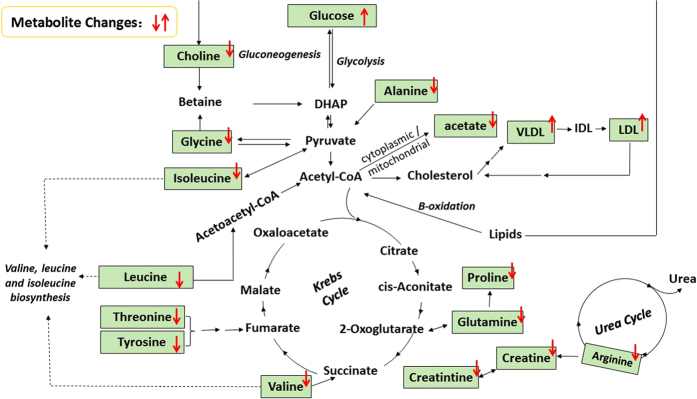
Schematic diagram of the perturbed metabolic pathways detected by ^1^H NMR analysis showing the interrelationship of the identified metabolic pathways. Red arrows (“↑↓”) in different colors represented the notable increase or decrease of metabolites in the serum.

In our study, four unique metabolic pathways of arginine and proline metabolism, glycine, serine and threonine metabolism, alanine, aspartate and glutamate metabolism, and pyruvate metabolism are identified from T2DM and CHD patients (Fig. 8). The altered metabolites related to T2DM-CHD are most involved in energy metabolism and amino acids metabolism (Fig. 9).

### Energy metabolism

Glucose is the major source material for ATP production in cells. ATP is mainly produced through metabolism of glucose under normoxia condition, which is composed of three relay pathways: citric acid cycle (TCA cycle, Krebs cycle), oxygen-independent pathway of glucose to pyruvate in cytoplasm and oxygen-dependent electron transfer chain, respectively^30^. It is expected that reduced oxygen level in CHD patients will significantly affect the TCA cycle since it is oxygen dependent. The anaerobic glycolysis begins to play a dominant role for ATP production under the conditions of hypoxia, leading to the disorder of glucose.

Creatine, synthesized in the liver and kidney, is transported through the blood and taken up by tissues with high energy demands. It can reflect the changes of energy metabolism in the muscles. Creatinine is derived from creatine and phosphocreatine. Creatine has the ability to increase muscle stores of phosphocreatine, potentially increasing the muscle’s ability to resynthesize ATP from ADP to meet increasing energy demands. Therefore, the level of creatine and creatinine also reflect the disorder of energy metabolism in T2DM-CHD patients.

### Amino acids metabolism

Leucine, isoleucine and valine are essential amino acids whose carbon structures are marked by branch points (BCAA). These three amino acids are critical to human life and are particularly involved in stress, energy and muscle metabolism. BCAA, especially leucine, can be an important source of calories, and is superior as fuel to the ubiquitous intravenous D-glucose, and it also can stimulate insulin released by pancreatic b-cells *in vitro*^31^. As important insulin secretagogues, BCAAs exert a regulatory effect on proteolysis and participate in building body organs^32^. Altered BCAA metabolism is one of the characteristics of T2DM.

As the most abundant amino acid in the serum, glutamine is the most important amino acid gluconeogenic precursor for adding new carbon to the glucose pool^33^. Turer *et al*.^34^ used metabolomic profiling to compare cardiac extraction and plasma substrates, and demonstrated that patients with CHD had decreased concentration of glutamate/glutamine. Alanine is highly concentrated in muscle and is one of the most important amino acids released by muscle, functioning as a major energy source. It is an important participant as well as regulator in glucose metabolism, and its levels always parallel blood sugar levels. And reduced concentrations of glutamine and alanine were also observed in T2DM patients, which illustrated the enhancement of gluconeogenesis in the diabetic state. Some of the amino acids are associated with insulinopenia and thus would be seen to be a normal response to gluconeogenesis. Our results are consistent with previous studies which indicate that the conversion of glutamine and alanine is high in T2DM patients^35^. Karsten Suhre *et al*. reported that BCAA (leucine, isoleucine, and valine) all increased in the diabetes group^36^, and elevated levels of BCAA have been found up to 13.5 years ahead of clinical manifestation in the study of Walford *et al*., Floegel *et al*. and Wang *et al*.^37^,^38^,^39^, however, there is growing evidence that elevated BCAA levels may reflect a state of insulin resistance that is not necessarily specific to T2DM^40^.

Arginine is one of the most versatile amino acids in animal cells, serving as a precursor for the synthesis not only of proteins but also of nitric oxide, urea, polyamines, proline, glutamate, creatine and agmatine^41^. It may stimulate the oxidation of energy substrates (including fatty acids and glucose) in adipocytes, liver, skeletal muscle, heart and whole body. Fu *et al*. have reported that dietary L-arginine supplementation markedly reduced white-fat mass in Zucker diabetic fatty rats^42^.

Isopropanol belongs to the family of alcohols and polyols compounds. The previous report indicated that isopropanol is one of the products from propanoate metabolism, and the substrate for synthesizing acetone catalyzed by the enzyme isopropanol dehydrogenase^43^. Alcohol dehydrogenase oxidizes alcohols to either aldehydes or ketones, with concomitant reduction of NAD^+^ to NADH^44^. Thus, we suggested that the isopropanol is associated with acetone metabolism, which may be a significant differential metabolite in T2DM.

For all we know, this study presented a holistic view of the metabolic changes related to T2DM-CHD and may contribute to its diagnosis. However, limitations of our study included a relatively small sample size in each group, which might prevent the differences in some metabolites from being fully apparent, and imperfect diagnostic approaches of altered metabolites. In addition, our understandings of these altered metabolites and their underlying mechanisms remain at rudimentary levels. Future work will focus on confirming/validating current metabolite findings in larger independent patient cohorts and elucidating the biological mechanisms.

## Conclusion

In the present study, ^1^H NMR-based metabolomics method combined with multivariate data analysis were used to distinguish independently T2DM-CHD patients from healthy controls with high reliability. About 17 potential biomarkers related to T2DM-CHD disease were found by analysis and 16 of the 17 metabolites used as the biomarkers in diagnosing T2DM-CHD disease exhibited a sensitivity of 92.9%, a specificity of 93.3% and an accuracy of 93.2%. This study has been proved to be useful in improving the diagnosis of T2DM-CHD which may provide new insights to identify additional novel biomarkers.

## Materials and Methods

### Ethical approval

All procedures were designed according to the Declaration of Helsinki’s ethical principles. The study protocol has already been ethically reviewed and approved by Ethics Review Committee of Beijing University of Chinese Medicine and the methods were carried out in accordance with the approved guidelines. Patients were aware of their involvement and signed a written informed consent agreeing to the use of the resulting information for medical publications.

### Subjects and participants

The study was conducted with the approval of the ethical committee of Beijing University of Chinese Medicine and all study participants have given informed consent for the investigation. A total of 71 participants from the affiliated Dongzhimen Hospital of Beijing University of Chinese Medicine were matched for age and gender and equally distributed into four study groups: (i) T2DM patients; (ii) T2DM-CHD patients; (iii) CHD patients; (iiii) Healthy subjects as controls (HC). Detailed data about four study groups are listed in Table 1.

Diagnosis of diabetes was according to American Diabetes Association criteria (2005) and Diagnosis criteria of CHD referred to the WHO standard criteria (1979). From January 2013 to December 2014, we consecutively recruited patients who had been referred to the outpatient clinic from the affiliated Dongzhimen Hospital of Beijing University of Chinese Medicine for treatment of diabetes and coronary heart disease. There were 15 volunteers of HC subjects from the medical examination center of Dongzhimen Hospital in the same period of time.

General information, past medical history, family history, personal history, and signs were collected within 24 hours after the patients were admitted. Details of information in the view of traditional Chinese four diagnostic methods were also recorded. Collections of patient histories and information from traditional four diagnostic methods were determined by the relevant professionals. Specific requirements for relevant professionals included having the occupation qualification, attending the physician or above, and having relevant clinical experience more than two years.

### Sample collection and preparation

Fasting blood samples were collected from the subjects in the morning by venipuncture and stored in EDTA-containing green-top tubes. Then the samples were centrifuged at 3 000 × g for 10 min at 4 °C to isolate plasma. The plasma samples were stored at −80 °C until further processing and analysis.

Plasma samples were thawed and prepared by mixing 200 μL of plasma with 400 μl of 1.5 M of deuterated phosphate buffer (NaH_2_PO_4_ and K_2_HPO_4_, including 0.1% TSP, pH 7.47), adding D_2_O up to 600 μL if the volume of serum is insufficient. The mixture was left to stand for 5 min at room temperature and then centrifuged at 13 000 rpm at 4 °C for 15 min. The supernatant solution (550 μL) was then transferred into a 5 mm NMR tube for NMR analysis.

### Acquisition of ^1^H-NMR spectra

All the samples were analyzed at 298 K using a VARIAN VNMRS 600 MHz NMR SPECTROMETER operating (Varian Inc, Palo Alto, Calif) at 599.871 MHz using a 5 mm inverse-proton (HX) triple resonance probe with z-axis gradient coil.

^1^H NMR spectra of plasma were recorded using the water-suppressed standard ^1^D CPMG pulse sequence (RD-90°-(τ-180°-τ)n-ACQ), where a fixed total spin-spin relaxation delay 2nτ of 320 ms was applied to attenuate the broad NMR signals from slowly tumbling molecules (such as proteins) and retain those from low-molecular weight compounds and some lipid components. The free induction decays (FIDs) were collected into 64 K data points with a spectral width of 12 000 Hz and 128 scans. The FIDs were zero-filled to double size and multiplied by an exponential line-broadening factor of 0.5 Hz before Fourier transformation (FT). Standard COSY, TOCSY, HMBC and J-resolved spectra were also acquired for metabolite identification purposes for the selected plasma samples.

### Data reduction and multivariate pattern recognition analysis

All of the ^1^H NMR spectra were manually phased and corrected for baseline distortion by MestReNova7.1.0 software (Mestrelab Research, Spain). All the spectra were referenced to the methyl group of lactate at δ1.336. In order to exploit all metabolic information embedded in the spectra, all NMR spectra (0.5–9.0) were segmented into equal widths of both 0.01 ppm and 0.001 ppm. Spectral regions of δ4.68–5.10, δ3.65–3.57, δ3.06–3.23, δ2.66–2.72 and δ2.53–2.60 were excluded to eliminate variations caused by imperfect water suppression, EDTA, and EDTA metal complexes. The area under the spectrum was then calculated for each segmented region and expressed as an integral value. The integrated data were normalized to the total sum of the spectrum before multivariate statistical analysis to give the same total integration value for each spectrum.

Subsequently, the integral values were imported into SIMCA-P+12.0 (Umetrics, Sweden) for multivariate statistical analysis. The data were mean centered for PCA and PLS-DA^45^,^46^,^47^, and in order to improve the separation due to groups and minimize other biological analytical variation, sample classes were modeled using the OPLS-DA algorithm at a unit variance scaled approach. The PCA and PLS-DA score plots were showed with the first principal component and the second principle component, while OPLS-DA were visualized with the first principle component and the first orthogonal component. The model coefficients locate the NMR variables associated to specific intervention as y variables. The model coefficients were then back-calculated from the coefficients incorporating the weight of the variables in order to enhance interpretability of the model; in the coefficient plot, the intensity corresponds to the mean-centered model (variance) and the color-scale derives from the unit variance-scaled model (correlation). Thus, biochemical components responsible for the differences between samples detected in the scores plot can be extracted from the corresponding loadings with the weight of the variable contributing to the discrimination. The coefficient plots were generated with MATLAB scripts (downloaded from http://www.mathworks.com) with some in-house modifications and was color-coded with absolute value of coefficients (r).

### Statistical analysis

Group means of metabolites’ integral are expressed as the mean ± SD. An independent sample T-test was used to detect significant differences in selected signals between the two groups by SPSS Statistics Base 17.0 (SPSS Inc, USA). P value less than 0.05 was considered to be statistically significant. Additionally, diagnostic model was constructed by the marker metabolites alone using linear discrimination analysis method. We used random forest clustering to interrogate the top biomarkers with significant alterations in the patients as compared to the control from the web site (http://www.metaboanalyst.ca/). The classification performance (sensitivity and specificity) of the OPLS-DA model and the area under the curve (AUC) of ROC were also calculated from the respective Monte-Carlo cross validation (MCCV) prediction (http://www.roccet.ca/).

## Additional Information

**How to cite this article**: Liu, X. *et al*. Identification of metabolic biomarkers in patients with type 2 diabetic coronary heart diseases based on metabolomic approach. *Sci. Rep*. **6**, 30785; doi: 10.1038/srep30785 (2016).

## Supplementary Material

Supplementary Information

## Figures and Tables

**Table 1:** Characteristics of Study Participants.

Variable	HC	CHD	T2DM-CHD	T2DM
N (male/female)	7/8	7/6	15/13	6/9
Age (years)	65.3 ± 6.6	64.7 ± 7.2	67.1 ± 5.8	63.9 ± 7.3
Duration of CHD	0	2.7 ± 4.9	3.4 ± 5.5	0
Duration of T2DM	0	0	5.5 ± 6.2	5.46 ± 4.1
BMI (kg/m2)	25.63 ± 2.04	26.02 ± 2.63	26.31 ± 2.62	24.98 ± 2.93
SBP (mmHg)	121.75 ± 10.65	122 ± 11.46	125.17 ± 12.02	113.64 ± 9.90
DBP (mmHg)	78.66 ± 9.58	75.67 ± 8.63	76.83 ± 11.13	77.10 ± 6.67
Triglycerides (mg/dL)	1.68 ± 0.19	1.95 ± 0.42*	2.12 ± 0.45*	1.34 ± 0.25
LDL (mmol/L)	3.26 ± 0.75	2.75 ± 0.83*	2.68 ± 0.75*	3.05 ± 0.59*
HDL (mmol/L)	1.30 ± 0.32	1.09 ± 0.29*	1.10 ± 0.25*	1.23 ± 0.28
Total cholesterol (mmol/L)	5.39 ± 0.44	6.04 ± 0.55	5.60 ± 0.13	5.74 ± 0.42
BUN (mmol/L)	4.40 ± 1.11	4.91 ± 1.02	5.70 ± 1.22	5.91 ± 1.41
SCr (mmol/L)	69.78 ± 14.04	68.44 ± 9.84	78.20 ± 15.89	65.32 ± 14.39
HbA1c (%)	6.96 ± 0.48	7.63 ± 1.92	8.81 ± 1.34*	8.59 ± 0.32*
FPG (mmol/L)	5.83 ± 1.97	5.34 ± 2.64	10.27 ± 3.29**	8.21 ± 2.94*
2hPG (mmol/L)	7.29 ± 1.24	6.61 ± 0.85	15.01 ± 4.26**	10.53 ± 4.53**
FINS (ulU/ml)	7.91 ± 3.56	8.96 ± 5.74	17.30 ± 9.33**	10.14 ± 6.91*
Medication History				
ACEI or ARB	0 (0)	6 (46.2)	18 (64.3)	0 (0)
Statins	0 (0)	4 (30.8)	11 (39.3)	0 (0)
Beta-blocker	0 (0)	5 (38.5)	10 (35.7)	0 (0)
OHA/insulin	0 (0)	0 (0)	13 (46.4)	3 (20.0)

BMI: body mass index; SBP: systolic blood pressure; DBP: diastolic blood pressure; LDL: low density lipoproteins; HDL: high density lipoproteins; BUN: blood urea nitrogen; SCr: serum creatinine; HbA1c: glycated hemoglobin; FPG: fasting plasma glucose; 2hPG: 2h plasma glucose; FINS: fasting insulin. Data are presented as mean ± SD. *p < 0.05, compared with control group, **p < 0.0001, compared with control group.

**Table 2:** Quantitative comparison of metabolites found in plasma of CHD patients, T2DM patients and healthy controls.

Metabolites	Chemical shift	Interal HC in group^a^ (mean ± std) × 10^−2^	CHD			T2DM		
			Interal in group^a^ (mean ± std) × 10^−2^	r^b^ (CHD vs. HC)	VIP	Interal in group^a^ (mean ± std) × 10^−2^	r^b^ (T2DM vs HC)	VIP
VLDL/LDL	**0.87(m)**, 1.27(m)	23.26 ± 4.11	28.81 ± 5.99**	0.52(↑)	2.06	21.04 ± 3.71	0.18(↓)	0.69
isoleucine	0.93(t), **0.99(d)**	7.42 ± 0.81	6.78 ± 1.20	0.23(↓)	0.79	5.85 ± 1.47**	0.56(↓)	1.87
valine	0.98(d), **1.03(d)**	15.8 ± 3.52	17.69 ± 4.28	0.10(↑)	0.28	10.42 ± 2.02**	0.71(↓)	2.26
isopropanol	**1.16(d)**	12.03 ± 6.77	1.57 ± 0.53**	0.73(↓)	3.08	1.33 ± 0.41**	0.73(↓)	2.51
alanine	**1.47(d)**	45.06 ± 8.74	36.07 ± 9.34*	0.44(↓)	1.92	27.19 ± 14.02**	0.60(↓)	2.06
leucine	**0.95(m)**, 1.70(m)	1.18 ± 0.42	0.74 ± 0.34**	0.56(↓)	2.13	0.66 ± 0.27**	0.64(↓)	2.00
arginine	1.90(m), **3.24(t)**, 3.76(t)	2.36 ± 0.41	1.68 ± 0.59**	0.58(↓)	2.41	1.59 ± 0.65**	0.54(↓)	1.97
acetate	**1.92(s)**	13.37 ± 3.7	10.0 ± 3.28*	0.49(↓)	1.86	11.74 ± 4.64	0.32(↓)	1.02
proline	2.03(m), **2.36(m)**, 3.43(m)	6.43 ± 0.81	7.91 ± 1.02**	0.48(↑)	1.44	4.58 ± 1.84**	0.53(↓)	1.85
glutamine	2.14(m), **2.46(m)**, 3.79(m)	3.62 ± 0.62	2.88 ± 0.60**	0.57(↓)	2.21	2.58 ± 0.91**	0.54(↓)	1.88
creatine	**3.02(s)**, 3.92(s)	3.46 ± 1.88	4.25 ± 1.92*	0.40(↑)	1.14	2.84 ± 1.98	0.39(↓)	1.46
creatinine	**3.03(s)**, 4.05(s)	19.92 ± 4.38	17.56 ± 3.08	0.20(↓)	0.94	13.42 ± 4.59**	0.60(↓)	1.99
glucose	3.24(m), **4.64(d)**, 5.23(d)	20.00 ± 5.99	23.13 ± 4.87	0.16(↑)	0.43	38.2 ± 22.42**	−0.53(↑)	1.65
glycine	**3.55(s)**	52.79 ± 11.87	42.61 ± 11.46*	0.41(↓)	1.72	55.02 ± 13.66	−0.58(↑)	1.96
threonine	1.31(d), 3.58(d), **4.25(m)**	4.35 ± 1.03	4.01 ± 1.50	0.03(↓)	0.06	2.63 ± 1.1**	0.66(↓)	2.12
tyrosine	6.89(m), **7.18(m)**	2.66 ± 0.47	2.75 ± 0.89	0.09(↑)	0.12	1.86 ± 0.92**	0.49(↓)	1.64
3-methylhistidine	7.00(s), **7.66(s)**	5.35 ± 1.55	3.94 ± 1.56*	0.44(↓)	1.78	4.70 ± 1.81	0.44(↓)	1.47

The arrows (↑/↓) were used to show the metabolite levels increase/decreased compared with healthy controls.

^a^The relative integrals of metabolites were determined from ^1^D ^1^H NMR analysis of plasma of each group.

^b^The values of correlation number extracted from the correlation plots of OPLS-DA models.

^c^The p values were obtained from student’s t-test. The chemical shifts in boldface were that we used in calculating integrals and p values.

**Table 3:** Quantitative comparison of metabolites found in plasma of T2DM-CHD patients and healthy controls.

Metabolites	HMDB	Chemical Shift	Interal in HC group^a^ (mean ± std) × 10^−2^	Interal in T2DM- CHD group^a^ (mean ± std) × 10^−2^	r^b^ (T2DM-CHD vs HC) (|r|> = 0.532)	VIP	p^c^ (T2DM-CHD vs HC) (p < 0.05)
VLDL/LDL	—	**0.87(m)**, 1.27(m)	23.26 ± 4.11	26.0 ± 6.27	0.176(↑)	0.84	0.135
isoleucine	HMDB00172	0.93(t), **0.99(d)**	7.42 ± 0.81	6.57 ± 1.01	0.487(↓)	1.45	0.008
valine	HMDB00883	0.98(d), **1.03(d)**	15.8 ± 3.52	13.39 ± 3.35	0.506(↓)	1.47	0.007
isopropanol	HMDB00863	**1.16(d)**	12.03 ± 6.77	1.63 ± 0.67	0.72(↓)	2.86	0.001
alanine	HMDB00161	**1.47(d)**	45.06 ± 8.74	30.20 ± 10.45	0.671(↓)	2.15	0.001
leucine	HMDB00687	**0.95(m)**, 1.70(m)	1.18 ± 0.42	0.79 ± 0.33	0.556(↓)	1.69	0.002
arginine	HMDB00517	1.90(m), **3.24(t)**, 3.76(t)	2.36 ± 0.41	1.52 ± 0.58	0.604(↓)	2.24	0.001
acetate	HMDB00042	**1.92(s)**	13.37 ± 3.7	9.64 ± 5.24	0.34(↓)	1.30	0.019
proline	HMDB00162	2.03(m), **2.36(m)**, 3.43(m)	6.43 ± 0.81	4.71 ± 1.61	0.583(↓)	1.87	0.001
glutamine	HMDB00641	2.14(m), **2.46(m)**, 3.79(m)	3.62 ± 0.62	2.49 ± 0.92	0.548(↓)	2.01	0.001
creatine	HMDB00064	**3.02(s)**, 3.92(s)	13.46 ± 3.88	9.76 ± 3.53	0.407(↓)	1.62	0.003
creatinine	HMDB00562	**3.03(s)**, 4.05(s)	19.92 ± 4.38	15.10 ± 4.06	0.51(↓)	1.79	0.001
glucose	HMDB00122	3.24(m), **4.64(d)**, 5.23(d)	20.0 ± 5.99	28.32 ± 7.83	0.536(↑)	1.78	0.001
glycine	HMDB00123	**3.55(s)**	52.79 ± 11.87	32.55 ± 7.75	0.72(↓)	2.64	0.001
threonine	HMDB00167	1.31(d), 3.58(d), **4.25(m)**	4.35 ± 1.03	3.00 ± 0.88	0.630(↓)	2.10	0.001
tyrosine	HMDB00158	6.89(m), **7.18(m)**	2.66 ± 0.47	2.03 ± 0.45	0.588(↓)	2.03	0.001
3-methylhistidine	HMDB00479	7.00(s), **7.66(s)**	5.35 ± 1.55	3.62 ± 1.37	0.55(↓)	1.85	0.001

The arrows (↑/↓) were used to show the metabolite levels increase/decreased compared with healthy controls.

^a^The relative integrals of metabolites were determined from 1D ^1^H NMR analysis of plasma of each group.

^b^The values of correlation number extracted from the correlation plots of OPLS-DA models.

^c^The p values were obtained from student’s t-test. The chemical shifts in boldface were that we used in calculating integrals and p values.
