# Supplementary material for: Identification of metabolic biomarkers in patients with type 2 diabetic coronary heart diseases based on metabolomic approach
Source: Sci Rep. 2016 Jul 29;6:30785. doi: 10.1038/srep30785 (PMC4965763; doi:10.1038/srep30785)
Supplement: Supplementary Information [file srep30785-s1.pdf]

Supplementary files

Identification of metabolic biomarkers in patients with type 2 diabetic coronary heart diseases based on metabolomic approach

Xinfeng Liu[1], Jian Gao[2], Jianxin Chen[2], Zhiyong Wang[2], Qi Shi[2], Hongxue Man[1], Shuzhen Guo[2], Yingfeng Wang[1], Zhongfeng Li[1,2,*] &Wei Wang[2,*]

1. Department of Chemistry, Capital Normal University, Beijing 100048, China.

2. Beijing University of Chinese Medicine, Beijing 100029, China.

Xinfeng Liu, Jian Gao and Jianxin Chen contributed equally to this work.

* Correspondence author:

Zhongfeng Li: Address: Department of Chemistry, Capital Normal University, No.105 Xisanhuanbeilu, Haidian District, Beijing 100048, China; E-mail: lizf@cnu.edu.cn; Tel: +86-10-68902655.

Wei Wang: Address: Beijing University of Chinese Medicine, No.11 Beisanhuandonglu, Chaoyang District, Beijing 100029, China; E-mail: wangwei@bucm.edu.cn; Tel: +86-010-64286508.

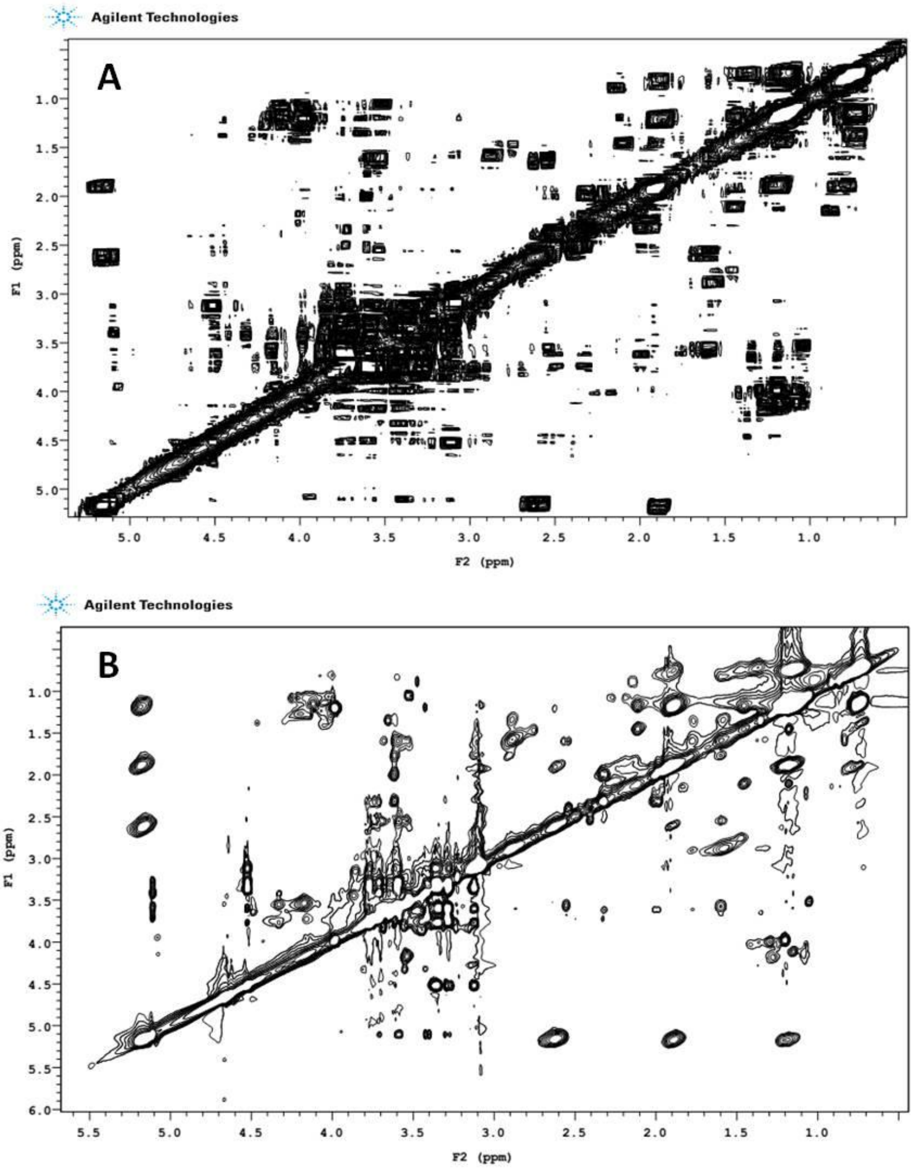

Figure. S1. NMR spectra of the aliphatic region of plasma sample (mixture of 30 metabolites) obtained with the following conditions: (A) gCOSY spectrum recorded in 5h with 32scans and 256 t1 increments. (B) TOCSY spectrum recorded in 11h with 64 scans and 256 t1 increments. All the samples were analyzed at 298 K using a VARIAN VNMRS 600 MHz NMR SPECTROMETER operating (Varian Inc, Palo Alto, Calif) at 599.871 MHz using a 5mm inverse-proton (HX) triple resonance probe with z-axis gradient coil.

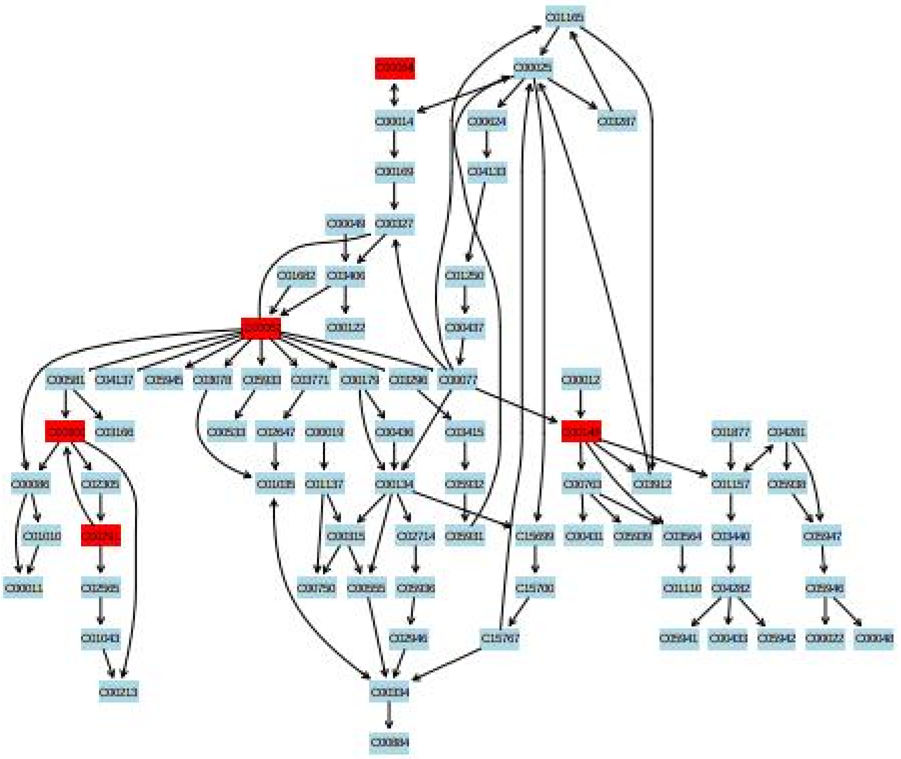

Figure S2. Pathways of Arginine and proline metabolism

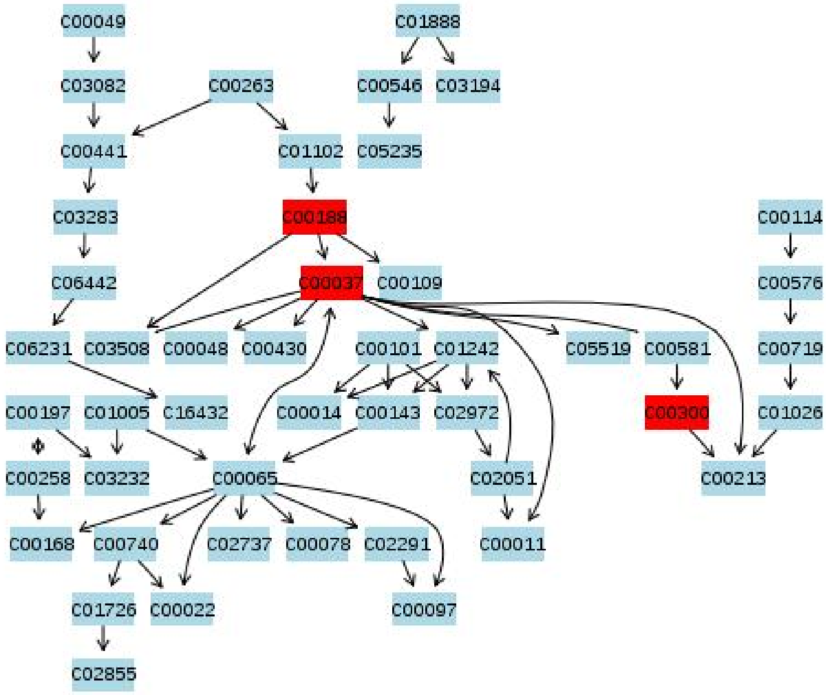

Figure S3. Pathways of Glycine, serine and threonine metabolism

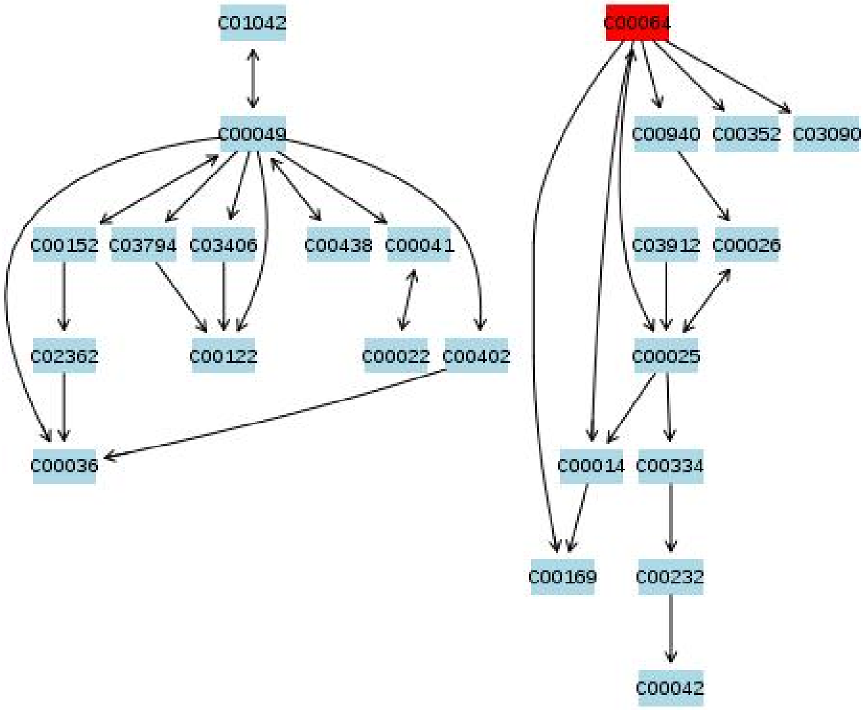

Figure S4 Pathways of Alanine, aspartate and glutamate metabolism

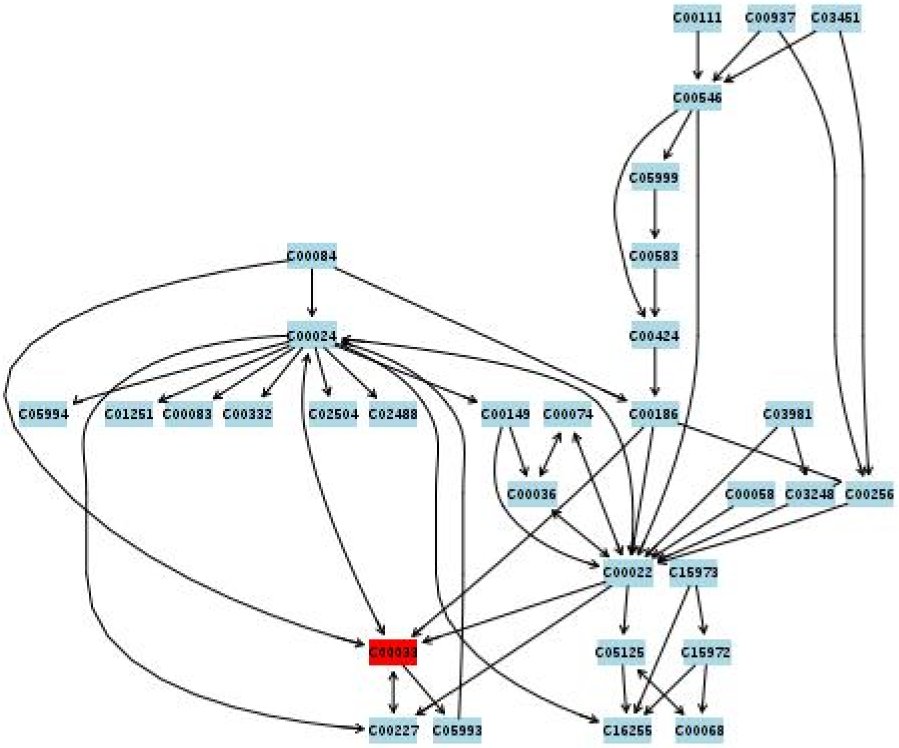

Figure S5. Pyruvate metabolism

Table S1. Results from Pathway Analysis with MetPA from plasma

Pathway Name	Total compounds	Expected	Hits	Raw p	FDR	impact	metabolites
Glycine, serine and threonine metabolism	48	0.31907	3	0.003474	0.05559	0.28506	threonine , creatine , glycine
Arginine and proline metabolism	77	0.51184	5	9.75E-05	0.0039	0.26671	glutamine, arginine, creatine, creatinine, proline
Alanine, aspartate and glutamate metabolism	24	0.34732	1	0.14857	0.66245	0.20703	glutamine
Pyruvate metabolism	32	0.21271	1	0.14857	0.66245	0.20703	acetate

Total is the total number of compounds in the pathway; the hits is the actually matched number from the user uploaded data; the raw p is the original p value calculated from the enrichment analysis; the impact is the pathway impact value calculated from pathway topology analysis.
